# Biomarkers Changes after Neoadjuvant Chemotherapy in Breast Cancer: A Seven-Year Single Institution Experience

**DOI:** 10.3390/diagnostics11122249

**Published:** 2021-11-30

**Authors:** Saverio Coiro, Elisa Gasparini, Giuseppe Falco, Giacomo Santandrea, Moira Foroni, Giulia Besutti, Valentina Iotti, Roberto Di Cicilia, Monica Foroni, Simone Mele, Guglielmo Ferrari, Giancarlo Bisagni, Moira Ragazzi

**Affiliations:** 1 Breast Surgery Unit, Azienda Unità Sanitaria Locale-IRCCS di Reggio Emilia, 42123 Reggio Emilia, Italy; saverio.coiro@ausl.re.it (S.C.); giuseppe.falco@ausl.re.it (G.F.); monica.foroni@ausl.re.it (M.F.); simone.mele@ausl.re.it (S.M.); guglielmo.ferrari@ausl.re.it (G.F.); 2 Breast Cancer Unit, Azienda Unità Sanitaria Locale-IRCCS di Reggio Emilia, 42123 Reggio Emilia, Italy; elisa.gasparini@ausl.re.it (E.G.); moira.foroni@ausl.re.it (M.F.); valentina.iotti@ausl.re.it (V.I.); roberto.dicicilia@ausl.re.it (R.D.C.); giancarlo.bisagni@ausl.re.it (G.B.); moira.ragazzi@ausl.re.it (M.R.); 3 Oncology Unit, Azienda Unità Sanitaria Locale-IRCCS di Reggio Emilia, 42123 Reggio Emilia, Italy; 4 Pathology Unit, Azienda Unità Sanitaria Locale-IRCCS di Reggio Emilia, 42123 Reggio Emilia, Italy; 5 Clinical and Experimental Medicine Ph.D. Program, University of Modena and Reggio Emilia, 41100 Modena, Italy; 6 Radiology Unit, Azienda Unità Sanitaria Locale-IRCCS di Reggio Emilia, 42123 Reggio Emilia, Italy; giulia.besutti@ausl.re.it

**Keywords:** ER, HER2, PR, ki67, breast cancer, immunohistochemistry, neoadjuvant therapy

## Abstract

The adoption of neoadjuvant chemotherapy (NACT) for breast cancer (BC) is increasing. The need to repeat the biomarkers on a residual tumor after NACT is still a matter of debate. We verified estrogen receptors (ER), progesterone receptors (PR), Ki67 and human epidermal growth factor receptor 2 (HER2) status changes impact in a retrospective monocentric series of 265 BCs undergoing NACT. All biomarkers changed with an overall tendency toward a reduced expression. Changes in PR and Ki67 were statistically significant (*p* = 0.001). Ki67 changed in 114/265 (43.0%) cases, PR in 44/265 (16.6%), ER in 31/265 (11.7%) and HER2 in 26/265 (9.8%). Overall, intrinsic subtype changed in 72/265 (27.2%) cases after NACT, and 10/265 (3.8%) cases switched to a different adjuvant therapy accordingly. Luminal subtypes changed most frequently (66/175; 31.7%) but with less impact on therapy (5/175; 2.8%). Only 3 of 58 triple-negative BCs (5.2%) changed their intrinsic subtype, but all of them switched treatment. No correlation was found between intrinsic subtype changes and clinicopathological features. To conclude, biomarkers changes with prognostic implications occurred in all BC intrinsic subtypes, albeit they impacted therapy mostly in HER2 negative and/or hormone receptors negative BCs. Biomarkers retesting after NACT is important to improve both tailored adjuvant therapies and prognostication of patients.

## 1. Introduction

The indications for neoadjuvant chemotherapy (NACT) in breast cancer have been implemented since 2006 [1]. Besides the traditional aims of tumor downsizing, downstaging and conservative surgery, the NACT setting has become an opportunity for in vivo assessment of a tumor’s response to standard treatment and newly developed therapy.

Thought to have equal survival rates as adjuvant cytotoxic chemotherapy, NACT could provide an important indicator of patient’s outcome since the pathological complete response (pCR) is a powerful prognostic predictor of better disease-free and overall survival, compared with the evidence of residual tumor after NACT [2,3,4,5]. Finally yet importantly, the adjuvant therapy regimen could be modulated based on response to NACT.

For these reasons, the adoption of NACT for breast cancer patients has substantially increased in our institution in the last few years. Both NACT and adjuvant therapies are mainly based on the assessment of three biomarkers on tumor tissue from preoperative biopsies worldwide: estrogen receptors (ER), progesterone receptors (PR) and HER2 status [6,7]. Ki67 is implemented as a fourth biomarker by the majority of European countries in this setting to subdivide tumors into five surrogated intrinsic subtypes with different prognosis and treatment implications: Luminal A-like, Luminal B-like HER2 negative, Luminal B-like HER2 positive, HER2-positive non-luminal and triple-negative [8]. The need to repeat these biomarkers after NACT is still a matter of debate, and currently, no univocal international guidelines regarding this topic have been introduced.

To date, in our institution, all four biomarkers have been systematically repeated in all cases of residual tumor after NACT.

Thus, the aims of this study were to verify in a large retrospective monocentric series of breast carcinoma undergoing NACT:

- the concordance/discordance rate of each tumor biomarker between pre-NACT biopsy and post-NACT surgical specimen;
- the effect of biomarker changes on intrinsic subtype;
- the impact of these changes in the “real-world” of adjuvant treatment, also in view of new literature evidence.

## 2. Materials and Methods

This retrospective study was performed in accordance with the Declaration of Helsinki and approved by the Institutional Board Review (Ethical Committee Number 305/2019/OSS/IRCCSRE).

### 2.1. Case Selection

The pathology and oncology databases were searched in our Institution (AUSL-IRCCS, Reggio Emilia, Italy) for patients with breast carcinoma who underwent NACT followed by breast surgery between 2011 and 2018. Patients with a pCR were excluded from the present study: pCR was determined according to the most widely accepted definition that considers the absence of residual invasive disease in the breast plus the absence of measurable disease in any axillary lymph node (ypT0N0); the possible presence of carcinoma in situ (ypTisN0) was also included in the definition since it has no impact in patient prognosis [9]. Tumor biomarkers were performed on both pre-NACT biopsy and post-NACT surgical specimens. Clinicopathological data were collected from electronic archives and included: patient sex and age, cancer stage, treatment type, tumor size, histologic subtype (NST, lobular, other), ER, PR, KI67 and HER2 status. Patients received NACT according to standard protocol of our Institution based on the current guidelines or within clinical trials as follows: 4–6 months of pre-operative treatment, based on anthracyclines and taxanes, with the addition of anti-HER2 targeted therapy if indicated.

### 2.2. Pathological Analysis

Tumor grade was assessed according to the Nottingham modification of the Bloom–Richardson (Elston and Ellis) system. Immunohistochemistry for ER (SP1, Ventana prediluted), PR (1E2, Ventana prediluted) and Ki67 (MIB-1, 1:100, Dako) was performed using standard protocols in an automated immunostainer (Ventana BenchMark, Tucson, AZ, USA); 3, 3′- diaminobenzidine was used as the chromogen and Harris’s hematoxylin as the counterstain. Testing of hormones receptors was performed following ASCO-CAP recommendations [10]. HER2 status was evaluated using frontline dual-color FISH (FDA-approved Vysis PathVysion Her-2 DNA probe kit, Abbott Laboratories, Abbott Park, IL, USA) as previously described [11] and reported in accordance with ASCO-CAP guidelines [12].

Tumors were classified according to the St. Gallen surrogate definition of intrinsic subtypes of breast cancer in [8]: luminal A-like (ER and PR positive/HER2 negative/Ki-67 < 20%), luminal B-like/HER2-negative (ER positive/HER2-negative/Ki-67 ≥ 20% or PR ≤ 20%), luminal B-like/HER2-positive (ER positive/HER2 positive), HER2-positive/non-luminal (ER and PR negative/HER2 positive) and triple-negative (TN) (ER, PR and HER2 negative).

Clinical and pathological stages were assessed according to the American Joint Committee on Cancer Staging (8th edition) [13].

### 2.3. Statistical Analysis

The results were analyzed in GraphPad Prism (v5.0, GraphPad Software Inc., San Diego, CA, USA) and SPSS Statics v22.0.

Statistically significant differences in tumor histotypes and grading pre- and post-NACT were assessed using the Pearson’s Chi-Squared test. A comparison of the biomarkers’ statuses between pre-NACT and post-NACT was performed using Fisher’s exact test and the Wilcoxon’s *t*-test. The correlation between intrinsic subtype change after NACT and clinicopathological features was evaluated using a *t*-test. Data were considered statistically significant with a *p*-value < 0.05. Regression analysis was also performed to further investigate a potential correlation between intrinsic subtype changes and age.

Concordance analysis of biomarkers status and intrinsic subtypes between pre- and post-NACT were assessed by Cohen’s Unweighted Kappa.

## 3. Results

### 3.1. Study Cohort

**Figure 1:**
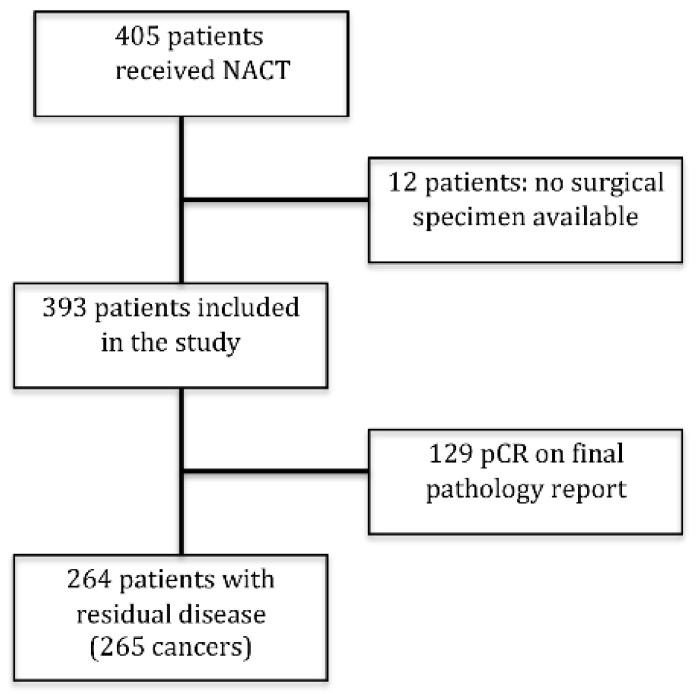
Case selection: among 405 consecutive patients undergoing NACT for breast cancer (January 2011–December 2018), 141 patients were excluded: 12 patients without surgical specimen (metastatic progression, surgery refusal, migration to another hospital), and 129 patients who had a pathological complete response (pCR). Therefore, 265 breast cancers from 264 patients (one patient had a bilateral tumor at presentation) were examined in the present study.

A total of 405 consecutive patients underwent NACT for breast cancer between January 2011 and December 2018 in our Institution. Patients whose surgical specimen was missing (metastatic progression, patient refusing surgery, migration to another hospital) (*n* = 12) and patients who had a pCR (*n* = 129) were excluded (Figure 1). Thus, we included in our study 264 patients with 265 carcinomas (one patient had bilateral synchronous tumors at presentation, both luminal B-HER2 positive). The patients were 263 females and 1 male, aged 25 to 86 years old (mean 53.88 ± 13.20). The clinical, histological and biological characteristics pre- and post-NACT are reported in Table 1.

The clinical stage at presentation was mainly stage II and III: 186 (70.2%) cases were stage II A/B, and 75 (28.3%) cases were stage III A/B. Of note, four (1.5%) cases of carcinoma stage IA were treated with NACT because of aggressive features. After NACT, cases were mainly stage I and II: 105 (39.6%) cases were stage I A/B, and 100 (37.7%) were stage II A/B. Overall, tumor downstaging occurred in 166 (62.6%) cases, whereas 40 (15.1%) cases showed progression, in the remaining cases tumor stage remained unmodified (data not shown).

After NACT, the majority of tumors were ypT1 (61.1%) and ypN0 (53.2%). Four cases showed a pT0 on the breast but a residual disease in lymph nodes; among them, three were triple-negative, and the other one was Luminal B Her2+. In these cases, residual disease in lymph nodes was characterized.

The invasive cancer histotype was NST in 244 (92.1%) cases, lobular in 16 (6.0%) cases, mixed NST and lobular in 3 (1.1%) cases, while the remaining 2 (0.8%) cases were 1 mucinous and 1 metaplastic carcinoma. After NACT, the tumor histotype was manly confirmed, except in five cases. Namely, one case of NST and one case of ILC were diagnosed as mixed after NACT, whereas three cases of mixed NST and lobular type carcinoma on biopsy showed only NST carcinoma on surgical specimen.

Concerning the pre/post-NACT biomarkers modification, we observed a slight reduction in ER mean expression (57.1% vs. 56.5%, *p* = 0.52) as an overall increase in ER-negative cases (77 vs. 84, *p* = 0.57), albeit not statistically significant. Contrariwise, a significant decrease in PR (31.0% vs. 19.6%, *p* = 0.001) and Ki67 mean expression (36.9 vs. 27.5, *p* = 0.001) were documented. Furthermore, a statistically significant increase in cases with low PR and Ki67 expression was observed: cases with PR < 20% were 146 pre-NACT and 186 post-NACT and cases with Ki67 < 20% were 46 pre-NACT and 144 post-NACT. Regarding HER2 status, after NACT, we observed a slight reduction in HER2 positive cases (105 vs. 91 cases, *p* = 0.13).

### 3.2. Change in Biomarkers Status

Changes in ER, PR and HER2 status (negative to positive or positive to negative) were observed in 31 (11.7%), 44 (16.6%) and 26 (9.8%) cases, respectively (Table 2). Among hormone receptors, progesterone receptors changed more frequently than estrogen receptors. Ki67 changed cut-off in 114 (43.0%) cases, becoming <20% in 106 (93.0%) cases. Thus, overall, a trend towards a negative shift was observed in hormone receptors and HER2, as well as a shift towards Ki67-low values post-NACT. Of note, 12 (4.5%) cases became ER-positive, 8 (3.0%) cases PR-positive, 8 (3.0%) cases Ki67-high and 6 (2.3%) cases HER2 positive. Concordance analysis demonstrated that HER2 was the most stable biomarker (*K 0.79*), whereas Ki67 was the most prone to change (*K 0.186*) from pre-NACT biopsy to post-NACT surgical specimen.

### 3.3. Change in Intrinsic Subtype and Therapy

Table 3 describes intrinsic subtype changes and their impact on adjuvant therapy.

At diagnosis, the majority of tumors were Luminal B-like Her2-negative (30.9%) and Luminal B-like Her2-positive (27.6%). Fifty-eight (21.9%) cases were triple-negative and 32 (12.1%) were HER2-positive/non-luminal. Twenty (7.5%) cases of luminal A-like carcinomas were treated with NACT because of advanced stage.

Overall, among all 265 carcinomas, the intrinsic subtype changed in 72 (27.2%) cases after NACT. Among these, 10 (3.8%) cases switched to a different adjuvant therapy, accordingly. Namely, in six cases, HER2-positivity was documented after NACT; thus, trastuzumab was added to the adjuvant chemotherapy protocol; in two cases of triple-negative carcinoma pre-NACT, endocrine therapy was performed as residual carcinoma showed hormone receptors expression; the remaining case was a triple-negative carcinoma that changed into luminal B-like HER2-positive and patient became eligible for both trastuzumab and endocrine therapy.

Among the 20 cases of luminal A-like carcinomas on biopsy, seven (35%) cases changed intrinsic subtype after NACT. All cases became luminal B-like due to loss of the PR expression. A concomitant increase in Ki67 rate was detected in one case. No impact on adjuvant therapy was observed.

Thirty-one (37.8%) luminal B-like HER2-negative carcinomas changed intrinsic subtype after NACT. Twenty-two cases became luminal A-like, five cases changed into luminal-like HER2-positive, and four cases had TN residual carcinoma. In the adjuvant setting, trastuzumab was added in the five luminal HER2-positive cases.

Among 73 cases of luminal B-like Her2-positive carcinomas, 28 (38.4%) changed intrinsic subtype after NACT. Overall, 19 cases lost HER2 positivity, becoming luminal A-like (11 cases) or luminal B-like (8 cases). In four cases, residual carcinoma was HER2-positive non-luminal; the remaining five cases showed only a significant reduction in the Ki67 rate (luminal A-like HER2-positive). Adjuvant therapy was therefore unaffected.

The pre-NACT HER2-positive non-luminal cancers changed the intrinsic subtype in three cases (9.4%), becoming luminal B-like HER2-negative in two cases and TN in one case. Both luminal-like cases had endocrine therapy added.

Among 58 TN cases, three (5.2%) residual carcinomas after NACT revealed a different intrinsic subtype: two cases showed ER expression becoming luminal B-like HER2-negative, and one case was luminal B-like HER2-positive. In all three cases, adjuvant therapy was changed by adding endocrine therapy, and trastuzumab was also added in the latter case.

The correlation between intrinsic subtype change after NACT and clinicopathological features, such as age, cT, stage, histotype, grade, tumor progression or downstaging, was not statistically significant (data not shown). A regression analysis showed no correlation between intrinsic subtype changes and age (R2: 0.000; F: 0.059, sign.: 0.808).

## 4. Discussion

Breast cancer is a heterogeneous disease exhibiting phenotypic and genetic heterogeneity [14].

Both spatial and temporal heterogeneity have been advocated to explain biomarkers change after NACT. Spatial heterogeneity refers to intratumoral diversifications of cancer cells that could impact the representativeness of the biopsied material, while temporal heterogeneity indicates the capability of tumor cells to switch phenotype through a therapy-induced survival-mechanism pressure. The frequency of these phenomena in daily routine in terms of biomarkers changes after NACT, and their real impact on therapy is still largely unknown. Several studies addressed this topic with different results [15,16,17,18,19,20,21,22,23,24,25,26,27,28], and although some authors recommend retesting of biological markers after NACT [15], its actual benefit is debated, with a pathology international working group not currently recommending it [28]. Major limitations of the current literature are that not all laboratories systematically repeated the biological characterization after NACT and very few of them tested Ki67 routinely because of the lack of standardization and reproducibility in its assessment.

Herein, we reported a retrospective consecutive series of 265 invasive breast cancer undergoing NACT, in which a residual tumor was detected. Since, in our institution, we systematically repeat the biological characterization of ER, PR, HER2 and Ki67, we could verify the frequency of each biomarker change, the impact on intrinsic subtype and on subsequent adjuvant therapy. All biomarkers changed with an overall tendency toward a reduced expression. Changes in progesterone receptor and Ki67 were statistically significant (*p* = 0.001), as already described in the literature [18].

Ki67 was the biomarker that most frequently changed after NACT, switching from high to low in 40% of cases and vice versa in a further 3% of cases. The proliferation rate is commonly reduced by NACT, and the post-treatment Ki67 index has been shown to correlate with the long-term outcome after both neoadjuvant endocrine and chemotherapy [29,30]. The update of the Ki67 Working group suggested that, in a neoadjuvant setting, the Ki67 status could be used for an early determination of endocrine therapy efficacy (on biopsy during NACT) to suggest a switch to chemotherapy in the event of an insufficient decrease in this biomarker. Moreover, the same authors concluded that the Ki67 assessment might be of value for a late determination (on surgical specimen post-NACT) of both endocrine and chemotherapy benefit, in order to decide for further therapy or not [31].

Progesterone receptor was the second most frequently switched biomarker (16.6%). Among a cohort of 446 patients retested after NACT, Ahn et al. reported little changes in the ER and HER2 status, whereas the PR status changed significantly. Furthermore, these authors demonstrated that negative conversion of PR correlated with decreased disease-free survival, thus representing a poor prognostic indicator [23].

HER2 was the most stable biomarker in our series, confirming the same results pre- and post-NACT in 90.2% of cases. Good concordance of the HER2 status is almost consistent throughout the literature, being HER2 amplification tested with FISH, as in our series, more concordant than HER2 expression measured with immunohistochemistry. A switch to a negative HER2 receptor up to 43% of the patients was reported when considering only HER2 positive breast cancers treated with trastuzumab [15,16]. The loss of HER2 positivity in the residual tumor was found to be associated with worse recurrence-free or disease-free survival; this change could therefore potentially indicate the need for further systemic therapy [32,33,34].

To date, consequences on adjuvant treatment reported in literature were mainly related to the switch of hormone receptors (HR) or HER2 status from negative to positive, allowing adding further therapy.

In 2015, recommendations from an international working group (BIG-NABCG) suggested indeed that a reassessment of hormone receptors and HER2 after neoadjuvant therapy should be considered only in some cases, such as negative results on pretreatment core biopsy and/or no response to therapy. Among a cohort of 83 cases, Xian et al. reported that 25 (30%) patients demonstrated changes in post-NACT biomarker status. Nevertheless, these changes impacted the patient management only in four (4.8%) patients that were hormone receptor and/or HER2 negative before NACT [21].

In our seven-year experience, among 265 carcinomas, we almost confirm these data. Intrinsic subtype changed in 72 (27.2%) cases after NACT, and 10 (3.8%) cases switched to a different adjuvant therapy accordingly. In our series, luminal subtypes were the subgroups most likely subject to changes (31.71%; 66/175). In particular, Luminal B HER2+, which could acquire PR expression lose HER2 positivity or lose all hormone receptors maintaining HER2 positivity. Despite the highest frequency of biomarkers changes, this group is, to date, the less likely to modify its adjuvant therapy.

The TN group was the most stable subtype, but all changes in tumor biomarkers had an impact on the patient treatment: two of them added endocrine therapy due to ER expression, one added both endocrine and trastuzumab due to ER and HER2 positivity.

Of note, regarding HER2-positive and TN breast cancer patients, an update on current ASCO guidelines endorsed a modulation in the adjuvant therapy even in the presence of residual tumor with unchanged intrinsic subtype [35].

The KATHERINE open-label phase III clinical trial showed that among patients with HER 2 positive early breast cancer who had residual disease after NACT, the risk of recurrence or death was 50% lower with adjuvant trastuzumab emtansine (T-DM1) than with trastuzumab alone [36].

The CREATE-X open-label, phase III trial recently demonstrated that adjuvant capecitabine should be considered for TN residual cancer since it was shown to increase disease-free survival [37]. Moreover, other trials are investigating the possible role of immunotherapy in this setting [38].

## 5. Conclusions

To conclude, we confirm in a large retrospective series that biomarkers’ changes after NACT impacted therapy in a small percentage of cases (3.8%), and all cases were HER2 negative and/or hormone receptors negative. On the other hand, our study demonstrated significant biomarkers’ changes occurring in all intrinsic subtypes of breast carcinomas and no statistically significant correlation was found between biomarkers’ changes after NACT and clinicopathological features were investigated. Therefore, we can conclude by suggesting that biomarkers retesting after NACT should always be considered to improve both tailored adjuvant therapies and prognostication of patients.

## Figures and Tables

**Table 1:** Clinicopathological features of the cohort (265 tumors from 264 patients) pre- and post-neoadjuvant chemotherapy.

**PRE-NACT**		**POST-NACT**		***p*-Value**
**FEATURES**	**RESULTS**	**FEATURES**	**RESULTS**	
**Stage**				NA
IA	4	IA	103	
IIA	93	IB	2	
IIB	93	IIA	76	
IIIA	59	IIB	24	
IIIB	16	IIIA	41	
		IIIB	4	
		IIIC	15	
**TNM**				
cT1c	5	ypT0	4 *	NA
cT2	183	ypT1	162	
cT3	63	ypT2	70	
cT4	14	ypT3	22	
		ypT4	6	
			
cN0	116	ypN0	141	
cN+	149	ypN1	71	
		ypN2	35	
		ypN3	15	
		ypNx	4	
			
**Histology**				
NST	244 (92.1%)		244 (92.1%)	0.988 °
ILC	16 (6.0%)		17 (6.4%)	
Metaplastic	1 (0.4%)		1 (0.4%)	
NST + ILC	3 (1.1%)		2 (0.8%)	
Mucinous	1 (0.4%)		1 (0.4%)	
**Grading**				
G1	0		3	0.158 °
G2	148		137	
G3	117		125	
**ER range (mean)**	0–100 (57.1 ± 43.8)		0–100 (56.5 ± 44.3)	0.20 ^
<1%	77		84	0.571§
≥1%	188		181	
**PR range (mean)**	0–100 (31.0 ± 37.9)		0–100 (19.6 ± 31.7)	**<0.0001 ^**
<20%	147		186	**0.0006 §**
≥20%	118		79	
**Ki67 range (mean)**	4–95 (36.9 ± 22.1)		0–90 (27.4 ± 27.3)	**<0.0001 ^**
<20%	46		144	**<0.0001 §**
≥20%	219		121	
**HER2**				
POS	105		91	0.15 §
NEG	160		174	

ER: estrogen receptor; PR: progesterone receptor; ILC: invasive lobular carcinoma; IND: indeterminate; NA: Not Assessed; NACT: neoadjuvant chemotherapy; NEG: negative; NST: carcinoma of non-special type; POS: positive. * cases with residual disease in lymph nodes. Significant *p*-values are in bold. ° Pearson’s Chi-squared; ^ Wilcoxon signed-rank test; § Fisher’s Exact Test.

**Table 2:** Changes in the status of biomarkers between pre-NACT biopsy and post-NACT resection.

Biomarkers (Cut-Off)	Pre-NACT	Post-NACT	N (%)	% of Concordance	K [0.95 CI]
ER (≥1%)	+	+	169 (63.8)	88.3	0.723 [0.632–0.815]
	−	−	65 (24.5)		
	+	−	19 (7.2)		
	−	+	12 (4.5)		
PR (≥1%)	+	+	119 (44.9)	83.4	0.67 [0.581–0.759]
	−	−	102 (38.5)		
	+	−	36 (13.6)		
	−	+	8 (3.0)		
PR (≥20%)	+	+	72 (27.2)	80.0	0.582 [0.481–0.682]
	−	−	140 (52.8)		
	+	−	46 (17.4)		
	−	+	7 (2.6)		
Ki67 (≥20%)	+	+	113 (42.7)	57.0	0.186 [0.073–0.299]
	−	−	38 (14.3)		
	+	−	106 (40.0)		
	−	+	8 (3.0)		
HER2	+	+	85 (32.1)	90.2	0.79 [0.714–0.887]
	−	−	154 (58.1)		
	+	−	20 (7.5)		
	−	+	6 (2.3)		

K of concordance: Cohen’s Unweighted Kappa.

**Table 3:** Intrinsic subtype changes and their impact on adjuvant therapy.

Pre-NACT		Post-NACT					Intrinsic Subtype Agreement	
Intrinsic Subtype	N° Cases (%)	No Change (%)	Yes Change (%)	Intrinsic Subtype (n)	Therapy Change (n)	Therapy Added	% of Concordance	K [0.95 CI]
Lum A	20 (7.5)	13 (65.0)	7 (35.0)	Lum B (7)	0	None	84.9%	0.323 [0.129–0.516]
Lum B HER2-	82 (30.9)	51 (62.2)	31 (37.8)	Lum A (22) Lum A HER2+ (2) Lum B HER2+ (3) TN (4)	0 2 3 0	None Trastuzumab Trastuzumab None	81.1%	0.54 [0.425–0.655]
Lum B HER2+	73 (27.6)	45 (61.6)	28 (38.4)	Lum A (11) Lum A HER2+ (5) Lum B HER2- (8) HER2+ (4)	0 0 0 0	None None None None	86.0%	0.62 [0.506–0.733]
HER2+	32 (12.1)	29 (90.6)	3 (9.4)	Lum B HER2- (2) TN (1)	2 0	Hormonal therapy None	97.4%	0.877 [0.788–0.967]
TN	58 (21.9)	55 (94.8)	3 (5.2)	Lum B HER2- (2) Lum B HER2+ (1)	2 1	Hormonal therapy Trastuzumab and Hormonal therapy	97.0%	0.913 [0.853–0.972]
Total (%)	265 (100)	193 (72.8)	72 (27.2)		10 (3.8)		72.8%	0.656 [0.588–0.724]

Lum A, Luminal A-like; Lum B HER2 -, Luminal B-like HER2-negative; Lum B HER2 +, Luminal B-like HER2-positive; HER2+, HER2-positive non-luminal; TN, Triple-negative. K of concordance: Cohen’s Unweighted Kappa.

## Data Availability

The data presented in this study are available on request from the corresponding author. The data are not publicly available due to ethical committee restrictions.
